# Stigma and quality of life in lung cancer patients: The mediating effect of distress and the moderated mediating effect of social support

**DOI:** 10.1016/j.apjon.2024.100483

**Published:** 2024-04-12

**Authors:** Hyewon Lim, Hyunmi Son, Gyumin Han, Taehwa Kim

**Affiliations:** a Pusan National University Hospital, Seo-gu, Busan, Republic of Korea; b College of Nursing, Pusan National University, Yangsan-si, Gyeongsangman-do, Republic of Korea; c Research Institute of Nursing Science, Pusan National University, Yangsan-si, Gyeongsangman-do, Republic of Korea; d Department of Pulmonology and Critical Care Medicine, Pusan National University Yangsan Hospital, Yangsan-si, Gyeongsangman-do, Republic of Korea

**Keywords:** Lung carcinoma, Stigma, Distress, Quality of life, Social support

## Abstract

**Objective:**

This study aimed to investigate the mediating effect of distress on the relationship between stigma and quality of life (QOL) in lung cancer patients, and to explore the moderated mediating effect of social support.

**Methods:**

A total of 184 individuals diagnosed with primary lung cancer participated in the study. Data on general and disease-related characteristics, stigma, distress, QOL, and social support were collected using a comprehensive structured questionnaire. Medical records were also utilized for an in-depth analysis of disease-related attributes. The data were meticulously analyzed using the SPSS PROCESS macro ver. 3.4 for detailed insights.

**Results:**

The findings elucidated a clear pathway whereby stigma negatively impacted patients' QOL through the mediating effect of distress. Interestingly, the extent of this impact was significantly influenced by the presence of friendship support, underscoring its unique moderated mediating role. Conversely, support from family and health care professionals did not demonstrate a significant influence in this context.

**Conclusions:**

These findings underscore the importance of addressing stigma and distress to improve the QOL of lung cancer patients. The study highlights the pivotal role of friendship support in moderating this relationship, suggesting the need for tailored interventions to strengthen social networks. These insights provide valuable guidance for developing more nuanced and effective patient support strategies in oncology care.

## Introduction

Lung cancer is the second most common cancer diagnosed in the world^1^ and in South Korea.^2^ Lung cancer is the leading cause of cancer death and is usually diagnosed in people age 65 or older.^3^ The two main types of lung cancer are small cell lung cancer and non–small cell lung cancer (85%), and the 5-year relative survival rate of lung cancer patients has gradually increased.^4^ As the survival rate improves with evolution of treatment,^2^^,^^5^ a primary concern of cancer patients has shifted to their quality of life (QOL) and psychological issues they encounter during cancer treatment.^6^^,^^7^ For patients with cancer, QOL is an important factor in treatment and recovery,^7^ and it affects their prognosis, treatment decisions, and survival.^8^ Psychosocial factors have been reported to have a greater impact on QOL in cancer patients than demographics or disease-related factors.^9^

Lung cancer patients have a higher incidence and severity of psychological distress than other cancer patients^10^ because people believe that lung cancer is “preventable” and caused by smoking even though it develops regardless smoking history.^11^ Such distress has been identified as being affected by stigma.^12^ Stigma is a social process that involves the experience or recognition of negative social judgments related to health; characteristics of experiencing stigma include being excluded, rejected, criticized, or devalued.^13^ Thus, stigma is particularly associated with distress such as depression, anxiety, guilt, shame, and criticism.^12^ The National Comprehensive Cancer Network (NCCN) of America recommends measuring distress as the sixth vital sign in cancer patients and emphasizes distress management.^14^ Most importantly, stigma and distress has a significant impact on QOL;^15^^,^^16^ therefore, managing both stigma and distress must be considered in caring for lung cancer patients.^15^

The benefits of social support for lung cancer have been reported,^17^ and the need for social support is well known. However, it remains unclear how social support is involved in reducing lung cancer stigma and improving the QOL of lung cancer patients experiencing stigma. Regardless of their smoking history, lung cancer patients experience stigma; thus, they tend not to disclose their illness to avoid stigma, and that often results in loss of financial or general support from other people.^18^ Consequently, they experience social isolation and difficulties in asking others for help,^16^^,^^18^ and such inappropriate or limited social interactions affect their QOL.^6^ Social support for cancer patients is primarily provided by family members, friends, and/or medical staff who are key sources in the support systems, and their support helps decrease the level of anxiety, depression, and stigma, and an increase QOL of cancer patients.^19^^,^^20^ The COVID-19 pandemic has exacerbated these challenges, as cancer patients face heightened distress and lockdowns have limited social services, pushing public health to prioritize urgent needs.^21^^,^^22^ In particular, patients with lung cancer have expressed significant concerns about mental health related to social isolation or anxiety, as well as cancer management related to alternatives-to-traditional patient care.^23^ This highlights the need for further research into the role of social support in lung cancer care, especially in the context of the COVID-19 pandemic.

Social support has been reported to have a moderating effect on the relationships between QOL and psychosocial problems, such as depression, anxiety, or stress.^24^^,^^25^ However, existing literature, including a study highlighting the burden felt by elderly patients receiving help from adult children,^26^ and research reveals varied psychological responses of cancer patients to support from family and friends,^27^ points to a complex and nuanced understanding of social support. Building on these findings, our study seeks to delve deeper into how different types of social support specifically influences the stigma-distress-QOL nexus in lung cancer patients.

**Fig. 1:**
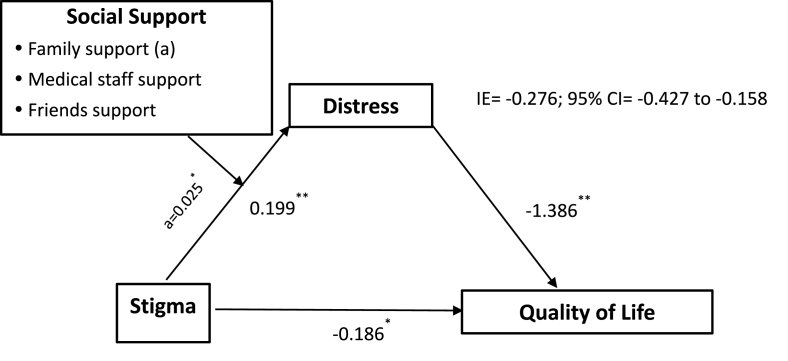
A visual representation of the moderated mediation model. IE = Indirect effects of the stigma-distress-quality of life relationship, a = effect of moderated mediation modeling. ∗*P* < 0.05, ∗∗*P* < 0.001.

The purpose of this study is to identify the mediating effect of distress in the negative relationship between stigma and QOL in lung cancer patients. Furthermore, we investigate the moderated mediation effect of social support in this process, specifically how social support affects the impact of stigma on QOL through distress (Fig. 1). We hypothesize that (1) stigma negatively correlates with QOL, (2) distress mediates the stigma-QOL relationship, and (3) the type and source of social support modulate the indirect effect of stigma on QOL via distress.

## Methods

### Design and participants

This study used a cross-sectional, descriptive design and self-reported questionnaire to test the hypothetical model. Participants were patients 18 years of age or older who had been diagnosed with primary lung cancer and received either inpatient or outpatient care. The participants were recruited from two university hospitals. This study included those who met the inclusion criteria and agreed to participate. Among these, individuals who were taking psychiatric medications or were receiving psychosocial interventions for their mental disorders were excluded. The multivariate normal distribution and estimation method^28^ was used to determine the number of participants. For the statistical power of the study, the number of independent variables 11 was multiplied by 15, based on the minimum ratio of 1:5 for the observed value and independent variables, and an appropriate ratio of 1:15 to 20. A total of 165 participants were calculated as the appropriate number of participants for this study. By considering a dropout rate of 20%, 200 participants were recruited, after excluding 16 of them who returned incomplete questionnaires the data of 184 participants were analyzed.

### Instruments

#### Sociodemographic and cancer characteristics

The items to measure sociodemographics were developed based on the previous studies associated with stigma in lung cancer patients^5^^,^^29^ including age, gender, education, average monthly income, number of family members living together, people living together, and smoking history. Cancer characteristics consisted of cancer stage at the time of diagnosis and treatment history. Cancer characteristics were collected by the researcher through medical records inquiries.

#### Stigma

Stigma was measured using the Lung Cancer Stigma Scale,^30^ which is the Korean version, the reliability and validity were verified by So et al.^31^ The 31 items include: 10 on social isolation, 11 on stigma and shame, five on discrimination, and five on smoking; all items are answered using a 4-point Likert scale. A minimum of 31 points and a maximum of 124 points, with a higher score indicating a higher stigma level. Cronbach's *α* of this scale was 0.96 at the time of tool development and 0.89 in the study by So et al.^31^ The reliability in this study was 0.92.

#### Distress

Distress was measured using the Hospital Anxiety and Depression Scale (HADS), developed by Zigmond and Snaith^32^ and translated into Korean by Oh et al.^33^ with verified reliability and validity. The HADS consists of seven questions on the anxiety subscale (HAD-A) and seven questions on the depression subscale (HAD-D), and each question is measured from 0 to 3 points. Each subscale ranged from zero to 21; higher scores reflect greater degrees of depression and anxiety. Cronbach's *α* at the time of development was 0.89. In the study by Oh et al.,^33^ Cronbach's *α* for anxiety and depression were 0.89 and 0.86, respectively.

#### Social support

The Multidimensional Scale of Perceived Social Support (MSPSS), originally developed by Zimet et al.^34^ on a seven-point Likert scale and verified for reliability and validity, was adapted into a five-point scale in the Korean version translated by Shin and Lee.^35^ This adaptation was used to measure an individual's perception of social support from three sources—friends, family, and medical staff. This tool consists of a total of 12 items in three sub-domains: four items for family, four items for friends, and four items for medical support. Each item is on a five-point Likert scale ranging from one point for ‘strongly disagree’ to five points for ‘strongly agree’. A higher score indicates a higher degree of support for each support system. Cronbach's *α* for each subscale was 0.86 for friend support 0.89 for family support, and 0.89 for medical staff support.

#### Quality of life

Quality of life was measured using the Korean version^36^ of Functional Assessment Cancer Therapy-General (FACT-G) version 4 developed by Cella and Tulsky.^37^ The FACT-G consists of 27 items on four subscales: seven on physical status, seven on social/family status, six on emotional status, and seven on functional status. Each item was measured from 0 points (not at all) to four points (very much), and higher scores signified a higher QOL. Cronbach's *α* at the time of development was 0.90 and 0.87 in the study by Kim et al.^36^ Cronbach's *α* in this study was 0.91.

### Data analysis

Data were analyzed using the SPSS/WIN 25.0 program.^38^ The general characteristics, disease-related characteristics, stigma, distress, QOL, and social support of the participants were identified using descriptive statistics. Pearson's correlation coefficient was used to analyze the correlations among the stigma, distress, QOL, and social support (friend support, family support, and medical staff support). The SPSS PROCESS macro (version 3.4) was used to analyze the mediating effect of distress on the relationship between stigma and QOL, the moderating effect of social support on the relationship between stigma and distress, and the moderated mediating effect of social support on the indirect effect of stigma on QOL through distress. For the moderation analysis, high, average, and low levels of the explanatory variables (stigma and social support) were defined using values of the means +1 standard deviation (SD), the means, and the means −1 SD, respectively. The Johnson–Neyman technique was additionally used to explore the conditions under which the moderating effect of social support was significant.

### Data collection

Data were collected from December 1, 2019 to June 30, 2020. Participants were patients who voluntarily indicated their willingness to participate in the study, and data collection were conducted after obtaining their written consent. In this study, medical professionals who understood the research objectives and procedures, were employed as research assistants. These assistants participated in the data collection together with the researchers, which included explaining the study's purpose, methods of participation, and procedures to the participants, as well as distributing and collecting questionnaires, and conducting medical record reviews. The study participants were recruited from the ward and outpatient departments. For outpatients, data were collected during the waiting time before treatment, and for inpatients, during hospitalization. In order for the subjects to answer comfortably, participants were asked to fill out the questionnaire in the outpatient waiting room for outpatients and in the education room or patient bed in the case of inpatient wards. With consent, information about the staging of cancer and treatment history was confirmed by researchers through medical records while the participant answered the questionnaire. Both researchers and research assistants directly handed out the questionnaires and collection envelopes to the participants. The completion of the survey took approximately 15–20 min, and a preselected gift was provided as a token of appreciation for participation.

### Ethical consideration

This study was conducted after obtaining approval from the Research Ethics Review Committee of the Pusan National University Hospital (IRB No. H-2001-006-087) and Pusan National University Yangsan Hospital (IRB No. 05-2019-189) Clinical Research Center Institutional Review Board. All participants provided written informed consent.

## Results

### Sociodemographic and cancer characteristics

Most participants were males (77.7%) and had a smoking history (81.0%). The average age of the participants was 65.29 (SD = 8.90) years. A majority of participants were diagnosed with non-small cell cancer (84.2%), and stage IV at the time of diagnosis was the most common among them. More characteristics of the participants were presented in Table 1.

**Table 1:** Demographic and disease-related characteristics of the sample (*N* = 184).

Characteristics	*n*	%
Age, years		
< 50	8	4.3
50–59	31	16.8
60–69	80	43.5
≥ 70	65	35.3
Gender		
Male	143	77.7
Female	41	22.3
Education		
Less than middle school	82	44.6
High school graduate	79	42.9
College graduate or more	23	12.5
Monthly income ($)		
< 2000	106	57.6
2000–3000	42	22.8
3000–4000	22	12.0
> 4000	14	7.6
Number of housemate (person)		
0	22	12.0
1	85	46.2
2–3	58	31.5
≥ 4	19	10.3
Type of housemate^a^		
Parents	8	3.1
Marriage partner	129	50.6
Brother or sister	7	2.7
Descendant	81	31.8
Others	8	3.1
Smoking history		
Never	35	19.0
Former	133	72.3
Current	16	8.7
Type and stage of cancer		
Non–small cell carcinoma	155	84.2
1 stage	23	12.5
2 stage	26	14.1
3 stage	41	22.3
4 stage	65	35.3
Small cell carcinoma	29	15.8
Limited stage	7	3.8
Extensive stage	22	12.0

Average of age was 65.29 (SD = 8.90).

a Duplicate response.

### Descriptive statistics and correlations among variables

The average of variables and the results of correlational analysis were illustrated in Table 2. Results showed that stigma was positively correlated with distress and negatively correlated with QOL but had no correlation with overall social support. Distress was negatively correlated with QOL and social support. Social support was positively correlated with QOL, even in correlation of the social support subscale, friend support was positively correlated with QOL as well as family support and medical staff support. However, friend support only showed a significant correlation with stigma and distress as well as QOL.

**Table 2:** Correlations among study variables statistics and correlations between variables.

Variable	Mean	SD	1	2	3	4
Stigma	51.85	13.97	–			
Distress	14.31	8.06	0.345∗∗	–		
Quality of life	64.67	17.24	−0.375∗∗	−0.700∗∗	–	
Social support	38.55	8.01	−0.134	−0.180∗	0.377∗∗	–
Friends support	12.58	3.88	−0.238∗∗	−0.264∗∗	0.362∗∗	–
Family support	16.20	3.72	−0.105	−0.049	0.329∗∗	–
Medical staff support	9.78	4.12	0.033	−0.091	0.167∗	–

∗*P* < 0.05.

∗∗*P* < 0.001.

### Mediation and moderated mediation models

The results of mediation and moderated mediation are shown in Fig. 1. The direct effect of stigma on QOL was statistically significant (β = −0.186, *P* = 0.007). There was also a significant indirect effect of stigma on QOL through distress: the bootstrapping results indicated an indirect effect (β = −0.276, 95% CI: −0.427, −0.158). The indirect effect accounted for 59.7% of the total effect, suggesting that distress partially mediated the association between stigma and QOL.

**Fig. 2:**
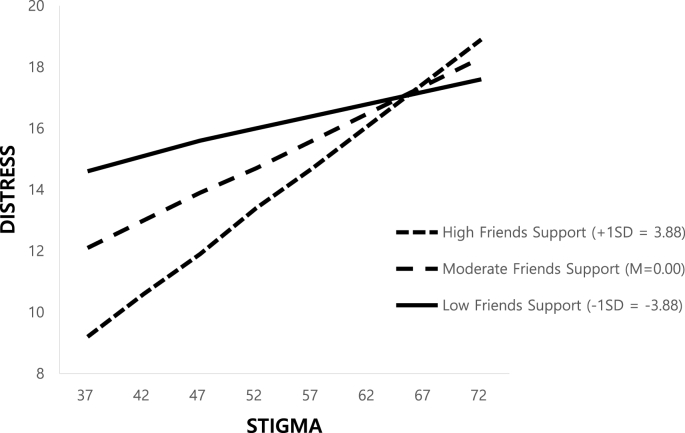
Friends support moderated the effect of stigma on distress.

As shown in Fig. 1, a test of moderation of the effect of stigma on distress yielded a significant result only by friend social support (β = 0.025, *P* = 0.043, 95% CI: 0.001, 0.049). On the other hand, the analysis indicated that there was no moderating effect of support from family β = 0.009, *P* = 0.443, 95% CI: −0.013, 0.030) or medical staff (β = −0.008, *P* = 0.397, 95% CI: −0.026, 0.011). We found that the effect of stigma on distress decreased as friend social support increased. There was a negative increasing trend in the coefficients of friend social support as its standard deviation moved from below to above the mean (Fig. 2). The moderated mediating index was −0.035 (bootstrap 95% CI: −0.0689, −0.0004), suggesting that the indirect effects of stigma on QOL of lung cancer patients through distress are moderated by friend social support. However, the moderated mediating effect was maintained when the stigma level was 65 points or lower (possible range value: 31–128 points) and increased in the Johnson–Neyman analysis when friend social support was 9.48 or higher (possible range value: 4–20 points).

## Discussion

This study revealed that stigma significantly impacts the QOL of lung cancer patients, with distress serving as a mediator. It also found that friend social support can mitigate the adverse effects of stigma on distress, suggesting the importance of supportive relationships in improving patient outcomes. These findings highlight the intricate relationship between psychological factors and social support in affecting the well-being of lung cancer patients.

This study was conducted to examine how social support affects the QOL of lung cancer patients experiencing stigma using a moderated mediation model of social support on the relationship between stigma and QOL in lung cancer patients through distress. Consistent with previous findings,^12^^,^^39^ we observed a significant mediating effect of distress on the relationship between stigma and QOL. This underlines that while stigma directly impacts QOL, this effect is also indirectly influenced by distress. In essence, stigma in lung cancer patients induces distress, leading to diminished QOL.

A significant moderated mediating effect of friend social support was observed in the relationship between stigma and QOL through distress. This finding aligns with other studies on breast cancer patients, where social support has been shown to alleviate anxiety and depression, thereby improving QOL.^7^^,^^40^ Similarly, research on elderly cancer patients indicates that strong social support from friends is beneficial for reducing depression and enhancing QOL.^41^ In our study, this pattern was also evident in lung cancer patients, averaging 65.3 years of age, with friend support moderating the stigma-distress-QOL dynamic. Consequently, this underscores the importance of cultivating robust friend social support networks for lung cancer patients. Given lung cancer's prevalence, especially among those aged 65 and older, enhancing friend support becomes crucial. Effective bolstering of this support could significantly diminish the adverse effects of stigma on these patients' QOL by mitigating distress.

However, our findings suggest a nuanced relationship where friend support seemingly amplifies distress levels in cases where the stigma score is high. This phenomenon can be attributed to the self-critical, shameful, and low self-esteem characteristics often associated with stigma, which foster social anxiety.^42^ Individuals grappling with these feelings tend to degrade their self-perception and shy away from social interactions, despite receiving positive feedback from others.^43^ Such behavior restricts their access to social support, adversely affecting their QOL.^43^^,^^44^ Echoing this, a study involving migrant women facing significant discrimination found that social support paradoxically led to negative outcomes like depression,^45^ resonating with our observation of heightened distress in the presence of strong friend support at high stigma levels. Consequently, this indicates the need for tailored strategies or diverse social support systems, adaptable to the varying degrees of stigma experienced by lung cancer patients.

In this study, neither family support nor medical staff support showed a significant moderating effect on the relationship between stigma and distress. Yet, it is notable that family support scored the highest, consistent with previous research.^46^^,^^47^ Family support is often characterized as strong and unconditional, distinct from other forms of support.^47^ However, this kind of support can sometimes feel burdensome, potentially diminishing a person's internalized sense of self-worth. This phenomenon was observed in a study where elderly patients reported feeling burdened when receiving help from their adult children.^26^ On the other hand, support from medical staff was reported as the lowest in our study, mirroring findings from earlier research.^46^ This lower level of support from medical staff might explain the absence of a significant moderating effect on our findings. Given that effective communication with medical staff has been shown to reduce stigma in lung cancer patients,^23^ future studies should consider investigating how enhanced communication with health care professionals might moderate the impact of stigma and distress.

### Implications for nursing practice and research

The results of this study showed that stigma mediated by distress influences the QOL of lung cancer patients. These results suggest that improving the QOL of lung cancer patients requires a change in the perception of stigma along with emotional support to address mental distress. Cognitive and emotional-behavioral interventions may be effective in this regard, as stigma is a subjective perception and interventions that address inappropriate perceptions can be beneficial. The current context of cognitive-behavioral therapy, which does not separate cognitive correction and emotional intervention supports the interpretation that addressing both cognition and emotion is more effective in improving QOL. This is in line with strategies that reinforce positive emotions, which focus not on changing incorrect cognition but on reducing negative emotions and enhancing positive ones, thereby allowing individuals to change their cognition through self-reflection. Moreover, regarding specific examples of nurse-led interventions, the most significant finding of this study is that peer support can regulate stigma. Nurses can enhance the effectiveness of related interventions by applying them through support groups and providing encouragement. When educating lung cancer patients, nurses can suggest ways to encourage social activities, such as clubs and hobbies. Especially in cases of high stigma levels, more careful strategies, such as counseling interventions, are needed to address stigma and psychological distress before the effects of social support can be expected.

### Limitations

Several limitations of this study should be considered. The outbreak of COVID-19 during our data collection posed challenges in recruiting outpatients for this study. Consequently, many subjects were those hospitalized for treatment, leading to an older age demographic. Most of the participants were older adults even though there was a trend of increasing incidence of lung cancer in young adults, so the data of social activities or incomes that can affect stigma and QOL were skewed. As young patients tend to actively engage in social activities, we suggest that further research should consider the age of the patient. This study utilized convenience sampling from two university hospitals, necessitating caution when generalizing the findings. Moreover, the data were collected using self-administered questionnaires, which may limit the validity of the results.

## Conclusions

In this study, pertinent to the field of oncology nursing, we systematically examined how stigma and distress interconnect to affect the QOL in lung cancer patients, with a specific focus on the role of social support from friends. Our data indicate that while distress serves as a crucial intermediary between stigma and QOL, the presence of friend support notably mitigates this link. However, we observed an unexpected increase in distress with higher levels of stigma despite friend support, pointing to a layered and complex dynamic. These findings highlight the need for oncology nursing professionals to develop and implement psychosocial interventions that are not only tailored to the unique needs of each lung cancer patient but also focus on enhancing social support networks, particularly in cases of high stigma levels. Such interventions may include facilitating support groups, encouraging social activities, and providing counseling services to address both stigma and psychological distress. This research contributes valuable insights into enhancing patient care in oncology nursing, advocating for the integration of strategic, and patient-centered psychosocial support to improve overall patient outcomes.

## Ethics statement

This study was conducted after obtaining approval from the Research Ethics Review Committee of the Pusan National University Hospital (IRB No. H-2001-006-087) and Pusan National University Yangsan Hospital (IRB No. 05-2019-189) Clinical Research Center Institutional Review Board. All participants provided written informed consent.

## Funding

This study received no external funding.

## CRediT authorship contribution statement

**Hyewon Lim:** Conceptualization or/and Methodology, Data curation or/and Analysis, Investigation, Resources or/and Software, Validation, Visualization, Writing original draft or/and review & editing. **Hyunmi Son:** Conceptualization or/and Methodology, Data curation or/and Analysis, Investigation, Project administration or/and Supervision, Resources or/and Software, Validation, Visualization, Writing original draft or/and review & editing. **Gyumin Han:** Conceptualization or/and Methodology, Data curation or/and Analysis, Resources or/and Software, Validation, Writing original draft or/and review & editing. **Taehwa Kim:** Conceptualization or/and Methodology, Data curation or/and Analysis, Investigation, Resources or/and Software, Validation, Writing original draft or/and review & editing. All authors had full access to all the data in the study, and the corresponding author had final responsibility for the decision to submit for publication. The corresponding author attests that all listed authors meet authorship criteria and that no others meeting the criteria have been omitted.

## Declaration of competing interest

The authors declare that they have no known competing financial interests or personal relationships that could have appeared to influence the work reported in this paper.

## Acknowledgments

The authors deeply appreciate all the patients participating in the study. We would like to acknowledge the support of the nurses, physicians, and decision-makers in the oncology department of hospitals who helped the study proceed despite the COVID-19 outbreak.

## Data availability statement

The data that support the findings of this study are available from the corresponding author, Hyunmi Son, upon reasonable request.

## Declaration of Generative AI and AI-assisted technologies in the writing process

No AI tools/services were used during the preparation of this work.

## References

[1] Sung H., Ferlay J., Siegel R.L. (2021). Global cancer statistics 2020: GLOBOCAN estimates of incidence and mortality worldwide for 36 cancers in 185 countries. CA A Cancer J Clin.

[2] (2023). Cancer statistics in Korea: incidence, mortality, survival, and prevalence in 2020. Cancer Res Treat.

[3] Bade B.C., Cruz C.S. (2020). Lung cancer 2020: epidemiology, etiology, and prevention. Clin Chest Med.

[4] Lu T., Yang X., Huang Y. (2019). Trends in the incidence, treatment, and survival of patients with lung cancer in the last four decades. Cancer Manag Res.

[5] Byun H., Kim E., Kim G. (2015). Impacts of stigma and distress on the quality of life in patients with lung cancer. Crisisonomy.

[6] Lee J.L., Kim K.S. (2011). The relationships between stigma, distress, and quality of life in patients with lung cancer. J Korean Oncol Nurs.

[7] Wildes K.A., Miller A.R., de Majors S.S.M., Ramirez A.G. (2009). The religiosity/spirituality of Latina breast cancer survivors and influence on health-related quality of life. Psycho-Oncology. J Psycholog Soc Behav Dimen Cancer.

[8] Seo J.Y., Yi M. (2015). Distress and quality of life in cancer patients receiving chemotherapy. Asian Oncol Nurs.

[9] Ha E.H., Lee S.H., Jeong J. (2010). Biopsychosocial predictors of the quality of life in breast cancer patients. J Breast Cancer.

[10] Batty G.D., Russ T.C., Stamatakis E., Kivimäki M. (2017). Psychological distress in relation to site specific cancer mortality: pooling of unpublished data from 16 prospective cohort studies. BMJ.

[11] Marlow L.A., Waller J., Wardle J. (2010). Variation in blame attributions across different cancer types. Cancer Epidemiol Prevent Biomark.

[12] Chambers S.K., Baade P., Youl P. (2015). Psychological distress and quality of life in lung cancer: the role of health-related stigma, illness appraisals and social constraints. Psycho Oncol.

[13] Weiss M.G., Ramakrishna J., Somma D. (2006). Health-related stigma: rethinking concepts and interventions. Psychol Health Med.

[14] Holland J.C., Bultz B.D. (2007). The NCCN guideline for distress management: a case for making distress the sixth vital sign. J Natl Compr Cancer Netw.

[15] Cataldo J.K., Jahan T.M., Pongquan V.L. (2012). Lung cancer stigma, depression, and quality of life among ever and never smokers. Eur J Oncol Nurs.

[16] Johnson L.A. (2019 May 1). Stigma and quality of life in patients with advanced lung cancer. Number 3/May 2019.

[17] Luszczynska A., Pawlowska I., Cieslak R., Knoll N., Scholz U. (2013). Social support and quality of life among lung cancer patients: a systematic review. Psycho Oncol.

[18] Conlon A., Gilbert D., Jones B., Aldredge P. (2010). Stacked stigma: oncology social workers' perceptions of the lung cancer experience. J Psychosoc Oncol.

[19] Finck C., Barradas S., Zenger M., Hinz A. (2018). Quality of life in breast cancer patients: associations with optimism and social support. Int J Clin Health Psychol.

[20] Shen M.J., Hamann H.A., Thomas A.J., Ostroff J.S. (2016). Association between patient-provider communication and lung cancer stigma. Support Care Cancer.

[21] Clifton K., Gao F., Jabbari J. (2022). Loneliness, social isolation, and social support in older adults with active cancer during the COVID-19 pandemic. J Geriatr Oncol.

[22] Adams-Prassl A., Boneva T., Golin M., Rauh C. (2022). The impact of the coronavirus lockdown on mental health: evidence from the United States. Econ Pol.

[23] Moraliyage H., De Silva D., Ranasinghe W. (2021). Cancer in lockdown: impact of the COVID-19 pandemic on patients with cancer. Oncologist.

[24] Huang C.-Y., Hsu M.-C. (2013). Social support as a moderator between depressive symptoms and quality of life outcomes of breast cancer survivors. Eur J Oncol Nurs.

[25] Yun E. (2014).

[26] Esbensen B.A., Østerlind K., Roer O., Hallberg I. (2004). Quality of life of elderly persons with newly diagnosed cancer. Eur J Cancer Care.

[27] Cicero V., Lo Coco G., Gullo S., Lo Verso G. (2009). The role of attachment dimensions and perceived social support in predicting adjustment to cancer. Psycho Oncol J Psycholog Soc Behav Dimensions Cancer.

[28] Hair JF, Anderson RE, Babin BJ, Black WC. Multivariate Data Analysis: A Global Perspective (Vol. 7).

[29] Brown Johnson C.G., Brodsky J.L., Cataldo J.K. (2014). Lung cancer stigma, anxiety, depression, and quality of life. J Psychosoc Oncol.

[30] Cataldo J.K., Slaughter R., Jahan T.M., Pongquan V.L., Hwang W.J. (2011). Measuring Stigma in People with Lung Cancer: Psychometric Testing of the Cataldo Lung Cancer Stigma Scale.

[31] So H.S., Chae M.J., Kim H.Y. (2017). Reliability and validity of the Korean version of the cancer stigma scale. J Korean Acad Nurs.

[32] Zigmond A.S., Snaith R.P. (1983). The hospital anxiety and depression scale. Acta Psychiatr Scand.

[33] Oh S.M., Min K.J., Park D.B. (1999). A study on the standardization of the hospital anxiety and depression scale for Koreans: a comparison of normal, depressed and anxious groups. J Korean Neuropsychiatr Assoc.

[34] Zimet G.D., Powell S.S., Farley G.K., Werkman S., Berkoff K.A. (1990). Psychometric characteristics of the multidimensional scale of perceived social support. J Pers Assess.

[35] Shin J.-S., Lee Y.-B. (1999). The effects of social supports on psychosocial well-being of the unemployed. Kor J Soc Welfare.

[36] Kim H., Yoo H.J., Kim Y.J. (2003). Development and validation of Korean functional assessment cancer therapy-general (FACT-G). Kor J Clin Psychol.

[37] Cella D.F., Tulsky D.S. (1993). Quality of life in cancer: definition, purpose, and method of measurement. Cancer Invest.

[38] Hayes A.F. (2018). Partial, conditional, and moderated moderated mediation: quantification, inference, and interpretation. Commun Monogr.

[39] Kim I., Kim S.Y. (2017). Stigma and quality of life in patients with lung cancer: the mediating effect of resilience. J Kor Data Inf Sci Soc.

[40] den Heijer M., Seynaeve C., Vanheusden K. (2011). Psychological distress in women at risk for hereditary breast cancer: the role of family communication and perceived social support. Psycho Oncol.

[41] Manning-Walsh J. (2005). Social support as a mediator between symptom distress and quality of life in women with breast cancer. J Obstet Gynecol Neonatal Nurs.

[42] Hamann H.A., Ostroff J.S., Marks E.G., Gerber D.E., Schiller J.H., Lee S.J.C. (2014). Stigma among patients with lung cancer: a patient-reported measurement model. Psycho Oncol.

[43] Heimberg R.G., Becker R.E. (2002).

[44] Akin S., Can G., Aydiner A., Ozdilli K., Durna Z. (2010). Quality of life, symptom experience and distress of lung cancer patients undergoing chemotherapy. Eur J Oncol Nurs.

[45] Ye J. (2017).

[46] Kim K.S., Yi M., Bang K.-S., Cho Y., Lee J.L., Lee E. (2013). Relationships among activity status, anxiety, depression, social support, symptom experience, and functional status in lung cancer patients based on the theory of unpleasant symptoms. Perspect Nurs Sci.

[47] Park L.E., Crocker J., Mickelson K.D. (2004). Attachment styles and contingencies of self-worth. Pers Soc Psychol Bull.

